# *catena*-Poly[[[bis­(thio­urea-κ*S*)cadmium]-di-μ-thio­cyanato-κ^2^ *N*:*S*;κ^2^ *S*:*N*] dihydrate]

**DOI:** 10.1107/S1600536812030267

**Published:** 2012-07-10

**Authors:** Anna Mietlarek-Kropidłowska, Jaroslaw Chojnacki

**Affiliations:** a Department of Inorganic Chemistry, Chemical Faculty, Gdansk University of Technology, 11/12 G. Narutowicza Str., 80-233 Gdańsk, Poland

## Abstract

The title compound, {[Cd(NCS)_2_(CH_4_N_2_S)_2_]·2H_2_O}_*n*_, forms a one-dimensional chain parallel to the *a* axis, caused by the presence of the bridging thio­cyanate groups. Two solvent mol­ecules per complex are present in the lattice. The Cd^II^ ion is situated on an inversion centre and is coordinated in a distorted octa­hedral fashion by two N and two S atoms from four thio­cyanate ligands and by two S atoms from two thio­urea mol­ecules. Weak O—H⋯S, N—H⋯O and N—H⋯N inter­actions reinforce the structure.

## Related literature

For a general introduction to thio­cyanato complexes, see: Nardelli *et al.* (1957 ▶). For the syntheses and structures of a series of cadmium complexes with thio­urea derivatives and thio­cyanato ligands, see: Wang *et al.* (2002 ▶); Cavalca *et al.* (1960 ▶); Zhu *et al.* (2000 ▶); Yang *et al.* (2001 ▶); Ahmad *et al.* (2008 ▶); Williams *et al.* (1992 ▶). For information on the properties of complexes incorporating these ligands, see: Yuan *et al.* (1997 ▶); Krunks *et al.* (1997 ▶); Amutha *et al.* (2011 ▶); Machura *et al.* (2011 ▶). For the use of Cd^II^ complexes with mixed S-donor ligands as precursors to CdS, see: Kropidłowska *et al.* (2008 ▶).
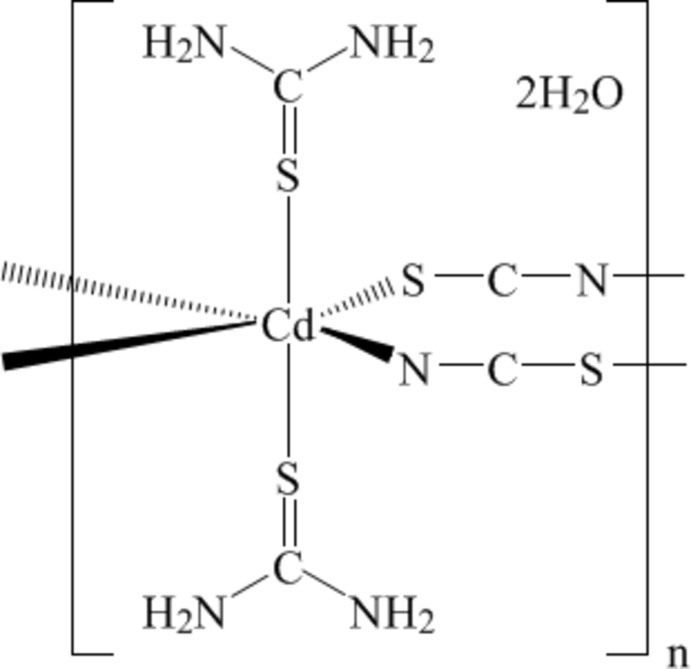


## Experimental

### 

#### Crystal data

[Cd(NCS)_2_(CH_4_N_2_S)_2_]·2H_2_O
*M* *_r_* = 416.84
Triclinic, 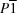

*a* = 5.8533 (3) Å
*b* = 7.3527 (3) Å
*c* = 8.8630 (4) Å
α = 73.413 (4)°
β = 76.926 (4)°
γ = 88.856 (4)°
*V* = 355.69 (3) Å^3^
*Z* = 1
Mo *K*α radiation
μ = 2.12 mm^−1^
*T* = 293 K
0.53 × 0.42 × 0.23 mm

#### Data collection

Oxford Diffraction KM-4-CCD diffractometer
Absorption correction: analytical [*CrysAlis PRO* (Oxford Diffraction, 2008 ▶), based on expressions derived by Clark & Reid (1995 ▶)] *T*_min_ = 0.558, *T*_max_ = 0.725
7642 measured reflections
2268 independent reflections
1985 reflections with *I* > 2σ(*I*)
*R*_int_ = 0.036

#### Refinement

*R*[*F* ^2^ > 2σ(*F* ^2^)] = 0.028
*wR*(*F* ^2^) = 0.062
*S* = 1.05
2268 reflections
85 parameters
3 restraints
H atoms treated by a mixture of independent and constrained refinement
Δρ_max_ = 0.47 e Å^−3^
Δρ_min_ = −0.51 e Å^−3^

### 

Data collection: *CrysAlis PRO* (Oxford Diffraction, 2008 ▶); cell refinement: *CrysAlis PRO*; data reduction: *CrysAlis PRO*; program(s) used to solve structure: *SHELXS97* (Sheldrick, 2008 ▶); program(s) used to refine structure: *SHELXL97* (Sheldrick, 2008 ▶); molecular graphics: *ORTEP-3* (Farrugia, 1997 ▶) and *Mercury* (Macrae *et al.*, 2006 ▶); software used to prepare material for publication: *WinGX* (Farrugia, 1999 ▶) and *publCIF* (Westrip, 2010 ▶).

## Supplementary Material

Crystal structure: contains datablock(s) I, global. DOI: 10.1107/S1600536812030267/fj2575sup1.cif

Structure factors: contains datablock(s) I. DOI: 10.1107/S1600536812030267/fj2575Isup2.hkl

Additional supplementary materials:  crystallographic information; 3D view; checkCIF report

## Figures and Tables

**Table 1:** Hydrogen-bond geometry (Å, °)

*D*—H⋯*A*	*D*—H	H⋯*A*	*D*⋯*A*	*D*—H⋯*A*
N1—H1*A*⋯O1^i^	0.86	2.26	3.049 (3)	153
N1—H1*B*⋯N3^ii^	0.86	2.3	3.147 (3)	167
N2—H2*A*⋯O1^i^	0.86	2.4	3.159 (3)	147
N2—H2*B*⋯O1^iii^	0.86	2.19	3.050 (3)	175
O1—H1*C*⋯S2	0.80 (2)	2.54 (2)	3.340 (2)	177 (4)
O1—H1*D*⋯S1^iv^	0.81 (2)	2.59 (2)	3.377 (2)	165 (3)

Symmetry codes: (i) 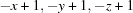; (ii) 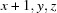; (iii) 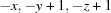; (iv) 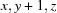.

**Fig. 1.**
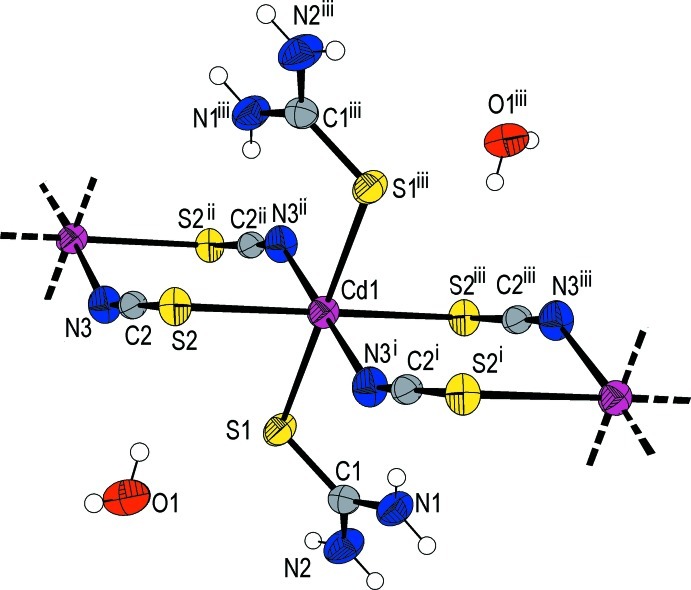
Molecular structure and atom-numbering scheme for [[Cd{SC(NH2)2}2(SCN)2].2H2O]n with displacement ellipsoids drawn at 50% probability level. H atoms are represented as arbitrary circles. Symmetry codes: (i) x+1, y, z; (ii) -x, -y+1, -z; (iii) -x+1, -y+1, -z.

**Fig. 2.**
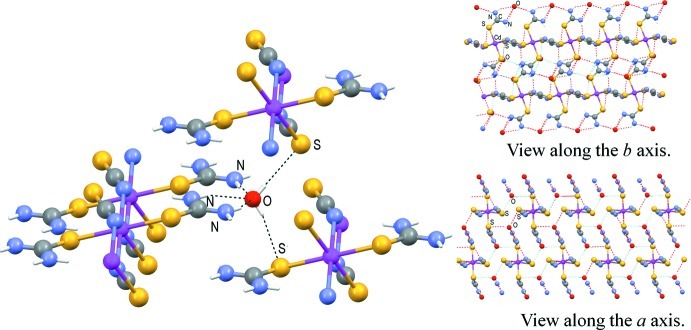
Weak interaction present in the crystal structure of [[Cd{SC(NH2)2}2(SCN)2].2H2O]n between the water and complex molecules (on the left). The arrangement of water and complex molecules in the crystal (on the right). Dashed lines denote the possible weak interactions.

## supplementary crystallographic information

### Comment

The interest in the coordination compounds possessing simultaneously thiourea and thiocyanato ligands dates back to the 1950s (*e.g.* Nardelli *et al.*, 1957) when the nature of coordination compounds formed by divalent cations (*M* = Mn, Co, Ni, Cd, Pb) and organic molecules containing sulfur was extensively studied. Besides the polymeric *catena*-poly[bis(thiocyanato-κ*N*)bis(µ-thiourea-κ^2^*S*:*S*)cadmium(II)] (Wang *et al.*, 2002), also structures of four other complexes with thiourea derivatives of [Cd(SCN)_2_(TU)_2_]_n_ type, where TU = ethylenethiourea (Cavalca *et al.*, 1960), *N*,*N*'-diphenylthiourea (Zhu *et al.*, 2000), *N*-phenylthiourea (Yang *et al.*, 2001; Ahmad *et al.*, 2008) or 1,3-dimethyl-2(3*H*)-imidazolethione (Williams *et al.*, 1992), can be found. The interest in these compounds is related either with their non-linear optical properties (Yuan *et al.*, 1997) or with the possibility to use them as single-source precursors of semiconducting materials based on CdS (Krunks *et al.*, 1997; Amutha *et al.*, 2011). Moreover, the use of SCN ligands, with bridging abilities, may lead to intriguing architectures and topologies, often generating one-dimensional chains (Machura *et al.*, 2011). It was the reason why, during our studies on the new molecular precursors (Kropidłowska *et al.*, 2008), we have turned our attention to systems of this type now and we have obtained a new complex containing thiourea and thiocyanato ligands connected to cadmium center.

The cadmium atom in *catena*-Poly[[[bis(thiourea-κ*S*)cadmium]-di-µ-thiocyanato-κ^2^*N*:*S*;κ^2^*S*:*N*] dihydrate] is located at the inversion center and is octahedrically coordinated by two S atoms and two N atoms from four thiocyanate groups as well as by two S atoms from thiourea molecules. The neighbouring Cd^II^ ions are bridged by two µ-SCN-κ^2^*N*:*S* ligands, thus forming eight-membered ring of [Cd-SCN]_2_ type with the Cd···Cd distance of 5.853 Å, which is close to the values observed in other bridged systems (Machura *et al.*, 2011). These units form one-dimensional chains of slightly distorted edge-shared Cd-centered octahedra along the [100] crystallographic direction. The Cd—S i Cd—N distances are typical for cadmium(II) thiocyanate complexes. The IR spectra clearly show the presence of the thiocyanato groups (with the maxima of ν_CN_ absorption at 2078 cm^-1^).

In the structure of [[Cd{SC(NH_2_)_2_}_2_(SCN)_2_]^.^2H_2_O]_n_, several weak interactions may be assumed, leading to the alternating arrangement of water and complex molecules. Each water molecule interacts with S or N atoms from the three neighboring polymeric chains. Thus, it can serve as a donor of a weak hydrogen bond to the sulfur atom from one of the thiourea moieties (O1(H1D)—S1^viii^) in one chain, as well as to sulfur from one thiocyanato ligand (O1(H1C) —S2) in the other. The oxygen lone pairs act as acceptors towards NH_2_ groups from thiourea moieties located within the third chain (N2(H2B) —O1^vii^, N1(H1A) —O1^v^, N2(H2A)—O1^v^). Finally, one "interchain" interaction, N1—H1B···N3^i^, operates between NH_2_ and SCN groups.

### Experimental

The reaction was carried out between 0.50 g cadmium(II) thiocyanate, Cd(SCN)_2_, and 1.34 g thiourea (molar ratio 1:8) which were dissolved in a small amount of water. The mixture was heated to 70°C and stirred using magnetic stirrer for 50 minutes and then left for crystallization at room temperature. After a few days two types of crystals appeared in the flask: needles (0.2 g) and blocks (0.1 g), which were mechanically separated under the microscope. The structure of needle-like crystals [Cd(SCN)_2_{µ-SC(NH_2_)_2_}]_n_ (m.p. 189°C) has been already described (Wang *et al.*, 2002), while the block-like crystals appeared to be a new compound, crystallizing as diaqua solvate [[Cd{SC(NH_2_)_2_}_2_(SCN)_2_].2H_2_O]_n_ (m.p. 187°C). The product, when taken from the mother liquor and dried using the filter paper, changes - becomes opaque and finally takes the form of a powder (most probably because of the removal of the solvent molecules). IR spectra were recorded using Mattson Genesis II Gold spectrometer equipped with Momentum Microscope as detector.

### Refinement

All N—H atoms were placed in calculated positions and refined as riding on their carrier atoms with N—H = 0.86 Å (NH_2_) and *U*_iso_(H) = 1.2 times *U*_eq_(N). Solvent O—H hydrogen atoms were found in the Fourier map and refined as constrained to: O–H bond length of 0.80 Å, H1C - H1D distance of 1.30 Å and *U*_iso_(H) = 1.5 times *U*_eq_(O) with the default uncertainties.

### Crystal data

**Table:**

[Cd(NCS)_2_(CH_4_N_2_S)_2_]·2H_2_O	*Z* = 1
*M**_r_* = 416.84	*F*(000) = 206
Triclinic, *P*1	*D*_x_ = 1.946 Mg m^−^^3^
Hall symbol: -P 1	Melting point: 460 K
*a* = 5.8533 (3) Å	Mo *K*α radiation, λ = 0.71073 Å
*b* = 7.3527 (3) Å	Cell parameters from 4676 reflections
*c* = 8.8630 (4) Å	θ = 2.9–33.8°
α = 73.413 (4)°	µ = 2.12 mm^−^^1^
β = 76.926 (4)°	*T* = 293 K
γ = 88.856 (4)°	Block, colourless
*V* = 355.69 (3) Å^3^	0.53 × 0.42 × 0.23 mm

### Data collection

**Table:**

Oxford Diffraction KM-4-CCD diffractometer	1985 reflections with *I* > 2σ(*I*)
Graphite monochromator	*R*_int_ = 0.036
ω–scan	θ_max_ = 31°, θ_min_ = 2.9°
Absorption correction: analytical [*CrysAlis PRO* (Oxford Diffraction, 2008), based on expressions derived by Clark & Reid (1995)]	*h* = −8→8
*T*_min_ = 0.558, *T*_max_ = 0.725	*k* = −10→10
7642 measured reflections	*l* = −12→12
2268 independent reflections	

### Refinement

**Table:**

Refinement on *F*^2^	Primary atom site location: structure-invariant direct methods
Least-squares matrix: full	Secondary atom site location: difference Fourier map
*R*[*F*^2^ > 2σ(*F*^2^)] = 0.028	Hydrogen site location: inferred from neighbouring sites
*wR*(*F*^2^) = 0.062	H atoms treated by a mixture of independent and constrained refinement
*S* = 1.05	*w* = 1/[σ^2^(*F*_o_^2^) + (0.0266*P*)^2^] where *P* = (*F*_o_^2^ + 2*F*_c_^2^)/3
2268 reflections	(Δ/σ)_max_ < 0.001
85 parameters	Δρ_max_ = 0.47 e Å^−^^3^
3 restraints	Δρ_min_ = −0.51 e Å^−^^3^

### Special details

**Table:**

Experimental. CrysAlisPro, (Oxford Diffraction, 2008). Analytical numeric absorption correction using a multifaceted crystal model based on expressions derived by (Clark & Reid, 1995).
Geometry. All s.u.'s (except the s.u. in the dihedral angle between two l.s. planes) are estimated using the full covariance matrix. The cell s.u.'s are taken into account individually in the estimation of s.u.'s in distances, angles and torsion angles; correlations between s.u.'s in cell parameters are only used when they are defined by crystal symmetry. An approximate (isotropic) treatment of cell s.u.'s is used for estimating s.u.'s involving l.s. planes.
Refinement. Refinement of *F*^2^ against ALL reflections. The weighted *R*-factor *wR* and goodness of fit *S* are based on *F*^2^, conventional *R*-factors *R* are based on *F*, with *F* set to zero for negative *F*^2^. The threshold expression of *F*^2^ > 2σ(*F*^2^) is used only for calculating *R*-factors(gt) *etc*. and is not relevant to the choice of reflections for refinement. *R*-factors based on *F*^2^ are statistically about twice as large as those based on *F*, and *R*- factors based on ALL data will be even larger.

### Fractional atomic coordinates and isotropic or equivalent isotropic displacement parameters (Å^2^)

**Table:**

	*x*	*y*	*z*	*U*_iso_*/*U*_eq_	
Cd1	0.5	0.5	0	0.03082 (8)	
S1	0.24899 (10)	0.38566 (9)	0.30122 (7)	0.03890 (15)	
S2	0.25651 (10)	0.82898 (8)	−0.03961 (7)	0.03377 (13)	
N1	0.6502 (3)	0.3430 (3)	0.3988 (3)	0.0424 (5)	
H1A	0.7326	0.2965	0.4686	0.051*	
H1B	0.7155	0.4193	0.3061	0.051*	
N2	0.3288 (4)	0.1803 (3)	0.5771 (3)	0.0481 (6)	
H2A	0.4141	0.1352	0.6452	0.058*	
H2B	0.1813	0.1488	0.6023	0.058*	
N3	−0.1946 (3)	0.6528 (3)	0.0619 (3)	0.0391 (5)	
C1	0.4240 (4)	0.2975 (3)	0.4340 (3)	0.0321 (5)	
C2	−0.0091 (4)	0.7257 (3)	0.0194 (3)	0.0281 (4)	
O1	0.1962 (4)	0.9249 (3)	0.3133 (2)	0.0524 (5)	
H1C	0.216 (7)	0.902 (5)	0.229 (3)	0.079*	
H1D	0.193 (7)	1.038 (3)	0.296 (4)	0.079*	

### Atomic displacement parameters (Å^2^)

**Table:**

	*U*^11^	*U*^22^	*U*^33^	*U*^12^	*U*^13^	*U*^23^
Cd1	0.02632 (12)	0.03241 (13)	0.03125 (13)	−0.00184 (9)	−0.00529 (9)	−0.00613 (10)
S1	0.0255 (3)	0.0545 (4)	0.0282 (3)	0.0010 (3)	−0.0048 (2)	0.0002 (3)
S2	0.0298 (3)	0.0265 (3)	0.0402 (3)	−0.0021 (2)	−0.0038 (2)	−0.0050 (2)
N1	0.0333 (10)	0.0557 (13)	0.0361 (11)	−0.0005 (10)	−0.0120 (9)	−0.0065 (10)
N2	0.0454 (12)	0.0560 (13)	0.0333 (11)	−0.0053 (11)	−0.0110 (10)	0.0044 (10)
N3	0.0330 (10)	0.0371 (11)	0.0500 (13)	−0.0009 (9)	−0.0106 (9)	−0.0158 (10)
C1	0.0357 (12)	0.0316 (11)	0.0295 (11)	0.0009 (9)	−0.0074 (9)	−0.0100 (9)
C2	0.0335 (11)	0.0264 (10)	0.0266 (10)	0.0050 (9)	−0.0101 (9)	−0.0088 (8)
O1	0.0662 (13)	0.0522 (11)	0.0412 (11)	−0.0028 (11)	−0.0197 (10)	−0.0113 (10)

### Geometric parameters (Å, º)

**Table:**

Cd1—N3^i^	2.3734 (19)	N1—H1A	0.86
Cd1—N3^ii^	2.3734 (19)	N1—H1B	0.86
Cd1—S1	2.6431 (6)	N2—C1	1.318 (3)
Cd1—S1^iii^	2.6431 (6)	N2—H2A	0.86
Cd1—S2^iii^	2.7585 (6)	N2—H2B	0.86
Cd1—S2	2.7585 (6)	N3—C2	1.154 (3)
S1—C1	1.714 (2)	N3—Cd1^iv^	2.3734 (19)
S2—C2	1.649 (2)	O1—H1C	0.797 (17)
N1—C1	1.317 (3)	O1—H1D	0.805 (17)
			
N3^i^—Cd1—N3^ii^	180	C1—S1—Cd1	111.15 (8)
N3^i^—Cd1—S1	95.01 (6)	C2—S2—Cd1	96.75 (7)
N3^ii^—Cd1—S1	84.99 (6)	C1—N1—H1A	120
N3^i^—Cd1—S1^iii^	84.99 (6)	C1—N1—H1B	120
N3^ii^—Cd1—S1^iii^	95.01 (6)	H1A—N1—H1B	120
S1—Cd1—S1^iii^	180	C1—N2—H2A	120
N3^i^—Cd1—S2^iii^	89.81 (5)	C1—N2—H2B	120
N3^ii^—Cd1—S2^iii^	90.19 (5)	H2A—N2—H2B	120
S1—Cd1—S2^iii^	92.043 (19)	C2—N3—Cd1^iv^	148.15 (19)
S1^iii^—Cd1—S2^iii^	87.957 (19)	N1—C1—N2	118.6 (2)
N3^i^—Cd1—S2	90.19 (5)	N1—C1—S1	122.51 (18)
N3^ii^—Cd1—S2	89.81 (5)	N2—C1—S1	118.87 (18)
S1—Cd1—S2	87.957 (19)	N3—C2—S2	179.5 (2)
S1^iii^—Cd1—S2	92.043 (19)	H1C—O1—H1D	108 (3)
S2^iii^—Cd1—S2	180.00 (3)		
			
N3^i^—Cd1—S1—C1	−43.20 (10)	N3^ii^—Cd1—S2—C2	30.81 (10)
N3^ii^—Cd1—S1—C1	136.80 (10)	S1—Cd1—S2—C2	−54.18 (8)
S2^iii^—Cd1—S1—C1	46.79 (9)	S1^iii^—Cd1—S2—C2	125.82 (8)
S2—Cd1—S1—C1	−133.21 (9)	Cd1—S1—C1—N1	22.3 (2)
N3^i^—Cd1—S2—C2	−149.19 (10)	Cd1—S1—C1—N2	−157.98 (17)

Symmetry codes: (i) *x*+1, *y*, *z*; (ii) −*x*, −*y*+1, −*z*; (iii) −*x*+1, −*y*+1, −*z*; (iv) *x*−1, *y*, *z*.

### Hydrogen-bond geometry (Å, º)

**Table:**

*D*—H···*A*	*D*—H	H···*A*	*D*···*A*	*D*—H···*A*
N1—H1*A*···O1^v^	0.86	2.26	3.049 (3)	153
N1—H1*B*···N3^i^	0.86	2.3	3.147 (3)	167
N2—H2*A*···O1^v^	0.86	2.4	3.159 (3)	147
N2—H2*B*···O1^vi^	0.86	2.19	3.050 (3)	175
O1—H1*C*···S2	0.80 (2)	2.54 (2)	3.340 (2)	177 (4)
O1—H1*D*···S1^vii^	0.81 (2)	2.59 (2)	3.377 (2)	165 (3)

Symmetry codes: (i) *x*+1, *y*, *z*; (v) −*x*+1, −*y*+1, −*z*+1; (vi) −*x*, −*y*+1, −*z*+1; (vii) *x*, *y*+1, *z*.
